# Flora of Vladimir Oblast, Russia: an updated grid dataset (1867–2020)

**DOI:** 10.3897/BDJ.9.e68046

**Published:** 2021-05-28

**Authors:** Alexey P. Seregin

**Affiliations:** 1 Lomonosov Moscow State University, Moscow, Russia Lomonosov Moscow State University Moscow Russia

**Keywords:** vascular plants, dataset, flora, Vladimir Oblast, Russia, occurrence

## Abstract

**Background:**

The dataset covers wild tracheophytes (native species, naturalised aliens and casuals) of Vladimir Oblast, Russia. It includes only one occurrence per species per grid square, thereby recently confirmed earlier records are not duplicated. Georeferences are based on the WGS84 grid scheme with 342 squares with areas ranging from 94.7 km^2^ in the northernmost part to 98.2 km^2^ on the southern boundary (5′ lat. × 10′ long.). Each occurrence is linked to the corresponding grid square centroid, therefore actual coordinates, habitat details and voucher information are unavailable. In late 2011, the earlier version of the dataset was used for the production of grid maps in the standard "Flora of Vladimir Oblast: checklist and atlas". Additional records, obtained during field excursions of 2012 and 2013, were fully included in the "Flora of Vladimir Oblast: grid data analysis". The stable version of the dataset with 123,054 grid records (as of 1867–2013) was published in GBIF in 2017.

**New information:**

Data obtained in the field during 2014–2020, as well as those extracted from recently published sources, were digitised, structured and finally published in GBIF in April 2021. The last update added 7,000 new grid records. Currently, "Flora of Vladimir Oblast, Russia: an updated grid dataset (1867–2020)" contains 130,054 unique occurrences of 1,465 vascular plant taxa (species, hybrids, species aggregates) from Vladimir Oblast and tiny parts of the adjacent areas. The average number of grid records has grown over the seven years from 363 to 380 species. The grid occurrences are largely based on the field studies by the author, performed during 1999–2020 (121,737 records), as well as on data extracted from the relevant literature, unpublished sources, herbarium collections and citizen science projects (8,317 records). The taxonomic backbone of the occurrence grid dataset follows the accompanying checklist dataset to ensure correct cross-linking of the names. As of April 2021, the dataset on the Vladimir Oblast flora represents the fourth largest dataset on vascular plants of Russia published in GBIF.

## Introduction

**Figure 1.**
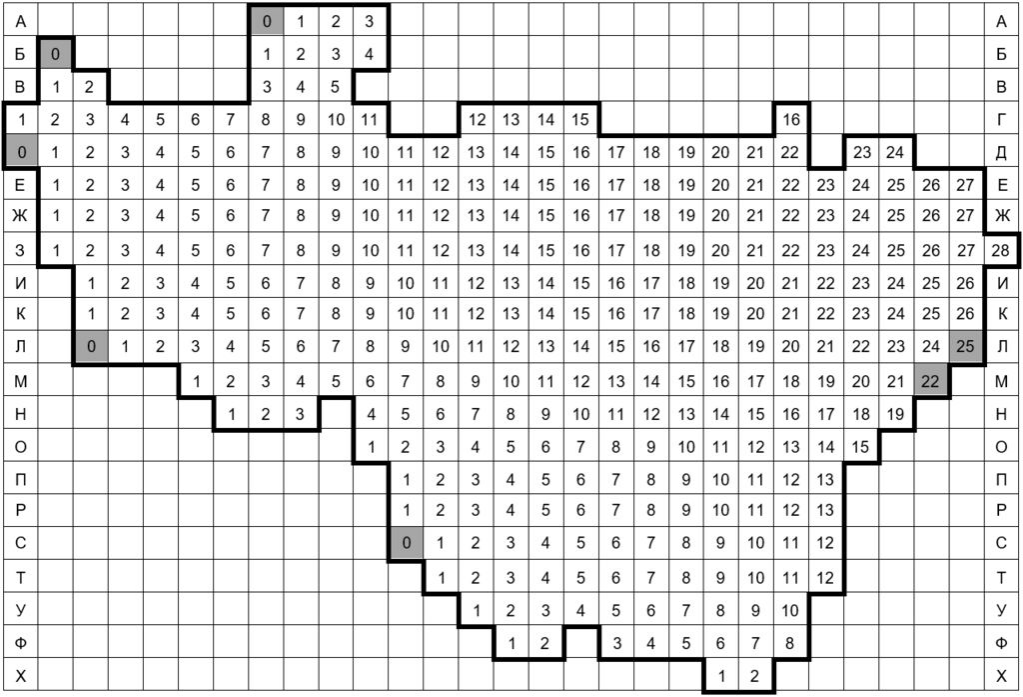
The grid scheme with 342 squares currently used for the floristic surveillance of Vladimir Oblast, Russia (5′ lat. x 10′ long.). There were 335 grid squares in the initial scheme (white squares). Subsequently, Seregin (2012) added **Л0** and **C0**, whereas Seregin (2014) added **A0** and **Д0**. In 2018, three grid squares (**Б0**, **Л25** and **М22**) were included for the better curation of some small areas of Vladimir Oblast outside the regular grid. The squares added during 2011–2018 are shown here in grey.

Since 1999, the author has been working on the grid mapping of the Vladimir Oblast flora. The region covers an area of 29,074 km^2^. The oblast was divided into 342 grid squares measuring 5′ lat. x 10′ long. or ca. 9.2 x 10.4 km. Thus, the area of the grid cells slightly increases southwards from 94.7 to 98.2 km^2^ (Fig. 1). Cyrillic letters were used to designate 21 rows from north to south, while numbers were used to indicate the squares within the rows from west to east. The northern border of the northernmost row **А** follows 56°50′N, while the southern border of the southernmost row **Х** follows 55°05′N, the western border of the squares **Г1** and **Д0** follows 38°10′E, while the eastern border of the square **З28** follows 43°00′E. The grid is available as a supplementary *.kml file (Suppl. material 1) with a copy on Zenodo (https://doi.org/10.5281/zenodo.4724913). The grid is visualised on Google Maps at https://maps.google.com/maps/ms?msid=200284766630468455543.000462414ec0fd70a9c6f&msa=0

Every year, data obtained by the author in the field were imported into the distribution database on the Vladimir Oblast flora (MS Excel spreadsheet). The earlier version of the database supplemented by all available records from the literature and herbarium collections was used to produce maps for the standard "Flora of Vladimir Oblast: checklist and atlas" (Seregin 2012). At the time of map production for the flora in November 2011, the database contained 118,231 records. In 2012–2013, the author continued the grid mapping of the Vladimir Oblast flora. By the end of 2013, the regional flora included 1,399 species of vascular plants (Seregin 2014). The stable version of the dataset with 123,054 grid records (as of 1867–2013) was published in GBIF in November 2017 (Seregin 2021b).

In line with the call for data papers describing datasets from Russia by GBIF, we completely revised the dataset and made the following improvements and ammendments:

1. Field data obtained by the author during 2014–2020 and new data published recently in various references were fully integrated into the dataset. New field data were obtained by the author during 77 standard one-day grid square surveys, as well as dozens of occasional field excursions focused on specific plant habitats, communities or species.
2. This update added 7,000 new grid records into the dataset, including records of 26 new species. For at least 11,190 grid records, the date of the last record was updated to show current presence of the species.
3. Three new grid squares were added on the fringes of Vladimir Oblast. The average number of grid records increased within seven years from 363 to 380 species (Table 1).
4. The taxonomic backbone of this occurrence dataset follows Seregin (2014), available in GBIF as a checklist dataset (Seregin 2021c) to ensure correct cross-linking of the names.
5. An aggregation of the records by standard grid square surveys was performed using the "eventID" field of the DarwinCore.

We amended the dataset on 29 Apr 2021 after a thorough data audit, performed by Dr Robert Mesibov (https://www.datafix.com.au) in line with preparation of the data paper.

As of 19 April 2021, the Vladimir Oblast occurrence dataset on the flora makes the seventh largest dataset on biodiversity of Russia published in GBIF (Table 2) and the fourth largest for vascular plants after Ueda (2021), Seregin (2021a) and Artemov and Egorova (2021). This is the only complete grid dataset for the first-level administrative divisions across Russia, although there are at least three GBIF-mediated datasets based on grid surveys of specific second-level administrative units in Tver, Saratov and Yaroslavl Oblasts of European Russia (Abramova and Volkova 2018, Frontova 2019, Pashkina 2019).

Amongst the datasets published by the Russian institutions, this occurrence dataset on the flora makes the fourth largest dataset available in GBIF (Table 3*^1^) and the third largest dataset for vascular plant diversity after Seregin (2021a) and Artemov and Egorova (2021).

## Project description

### Title

Grid mapping of the Vladimir Oblast flora

### Personnel

Alexey P. Seregin

### Study area description

Vladimir Oblast is located in Russia, specifically, in the central part of the East European Plain, ca. 100–400 km east of Moscow. It spans ca. 190 km from north to south and ca. 290 km from west to east, covering an area of 29,084 km^2^. The area is situated within the Volga River Basin with altitudes ranging from 67 to 271 m a.s.l.

**Climate**: The mean January temperature is −8.5°C, the mean July temperature is +18.7°C and the mean annual temperature is +4.7°C in the City of Vladimir. The mean annual precipitation level is 555 mm (ranging from 322 mm in 1967 to 783 mm in 2013) with the most precipitation occurring from June to November. Usually, snowcover lasts for 144 days from November to March with an average maximum snowdepth of 41 cm (Anonymous 2021). Continentality is more pronounced along the eastern border of Vladimir Oblast. According to the phenological data for the adjacent Moscow Oblast, the climatic conditions of which are similar to those in Vladimir Oblast, *Tussilago farfara* L. starts blooming on April 13 on average, superseded by *Alnus incana* (L.) Moench, *Daphne mezereum* L. and *Corylus avellana* L. from 16-18 April (Strizhev 1973). However, in the last decades, spring phenological events have been shown to begin earlier as compared to the long-term average values. For instance, *T. farfara* now starts blooming 21 days earlier than a century ago in the City of Kirov (Soloviev 2007).

**Vegetation and floristic divisions**: Vladimir Oblast is situated in the ecotone zone between boreal coniferous and temperate broadleaf (hardwood) forests. Distribution of the forest types within the region is clearly determined by the soil conditions. Both boreal coniferous forests dominated by *Pinus sylvestris* L. and *Picea abies* (L.) H. Karst. on various nutrient-poor substrata and temperate broadleaf forests with *Quercus robur* L., *Tilia cordata* L. and *Ulmus glabra* Huds. on loamy eutrophic soils being the main components of the original (pre-man) vegetation.

Other native plant communities of Vladimir Oblast are peat bogs, xeric meadows on steep slopes and alder stands along smaller streams, as well as meadows, marshes and willow thickets on flood plains. Currently, 29.9% of land is used for agriculture, while 55% is covered by forests (official data).

**Figure 2.**
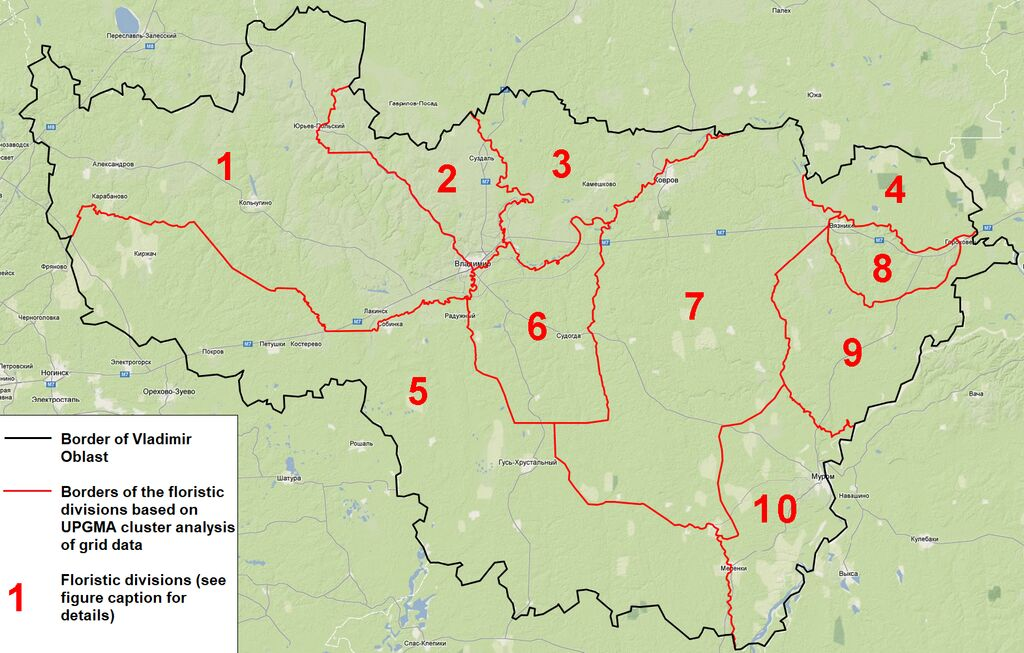
Floristic divisions of Vladimir Oblast, based on UPGMA cluster analysis of grid data (Seregin 2014) and species with the highest IndVal score (Dufrêne and Legendre 1997) within each division. Klin-Dmitrov Ridge (*Alnus incana* (L.) Moench)Opolye (*Phleum phleoides* (L.) H. Karst.)Nerl District (*Dactylorhiza fuchsii* (Druce) Soó)Balakhna (Frolishcheva) Lowland (*Jurinea cyanoides* (L.) Rchb.)Meshchera Lowlands (*Viola palustris* L.)Sudogda Upland (*Lamiastrum galeobdolon* (L.) Ehrend. & Polatschek)Oka-Tsna Ridge (*Salix rosmarinifolia* L.)Gorokhovets Ridge (no counts due to small area of the division and low number of corresponding grid squares)Lower Oka District (*Erigeron annuus* (L.) Pers. s. str.)Oka Plain (*Anthyllis macrocephala* Wender.)

Floristic divisions of Vladimir Oblast are based on UPGMA cluster analysis of grid data (Fig. 2) (Seregin 2014). This scheme corresponds, to some extent, to landscape divisions. Balakhna Lowland is the most distinct Region with Pyrolo–Pinetea forests and Oxycocco–Sphagnetea peat bogs. Three spatially-separated divisions (Meshchera Lowlands, Nerl District and Lower Oka District) have similar flora and vegetation consisting of Vaccinio–Piceetea boreal coniferous woods and various wetland vegetation.

In contrast, the Oka-Tsna Ridge with similar boreal forests typically lacks species from wetland habitats due to the proximity of limestone. The Klin-Dmitrov Ridge has the most eutrophic conditions and is characterised by the Querco–Fagetea and Galio–Urticetea classes, while, in the adjacent Opolye Querco–Fagetea, woodlands are framed by Trifolio–Geranietea sanguinei communities. The Gorokhovets Ridge and the Oka Plain are covered by Querco–Fagetea woods and xeric meadows with some diagnostic species of the Festuco–Brometea class. The Sudogda Upland is the only Region where both Querco–Fagetea and Vaccinio–Piceetea communities are equally present.

## Sampling methods

### Study extent

**Figure 3.**
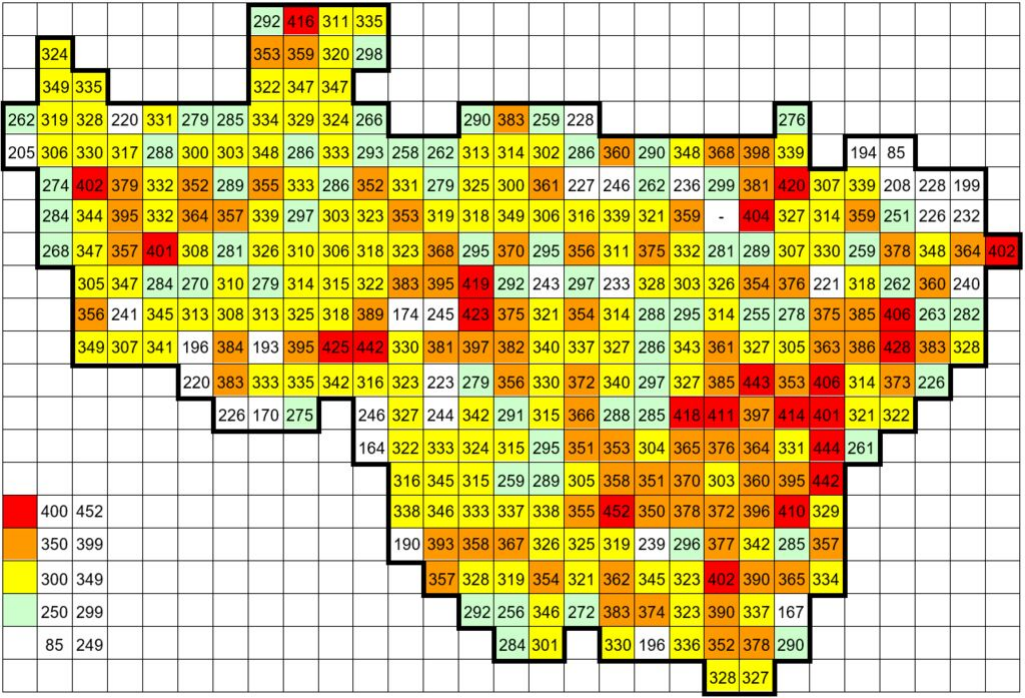
Numbers of grid records obtained during the most comprehensive one-day standard grid surveys (equalling the number of taxa).

The dataset combines two types of records, namely, field records by the author and data from other sources. The field records collected by the author (121,737 ocurrences) were obtained during 594 standard grid surveys. Typically, two surveys were performed in each grid square: (1) a summer survey (between June and September) and an additional (2) spring survey (late April to May). The numbers of grid records, obtained during the most comprehensive one-day standard grid surveys, are given on the map (Fig. 3).

Data extracted from the relevant literature, unpublished sources, herbarium collections and citizen science projects are not massive (8,317 records), since the dataset comprises only the latest records per grid for each species. A short historical overview of the most important sources was published in Russian in Seregin (2012) and Seregin (2014). Additionally, we integrated data from the citizen science project "Flora of Vladimir Oblast" (https://www.inaturalist.org/projects/vladimir-oblast-flora), initiated by the author on iNaturalist as part of the "Flora of Russia" initiative (Seregin et al. 2020). Surprisingly, the number of new grid records from the community was fairly modest. Only 959 occurrences out of 19,239 (as of 29 March 2021) were identified as new grid records, whereas another 200 occurrences accounted for recent confirmations of historical records.

### Sampling description

A standard one-day survey began with the preparation of the route using satellite images. It was designed to link known localities of rare species and areas of potential interest. Route planning helps to avoid various delays and fruitless searches. Plants that are difficult to identify in the field were collected for further examination as herbarium specimens. Previously-known localities of rare species were to be revisited.

**Figure 4.**
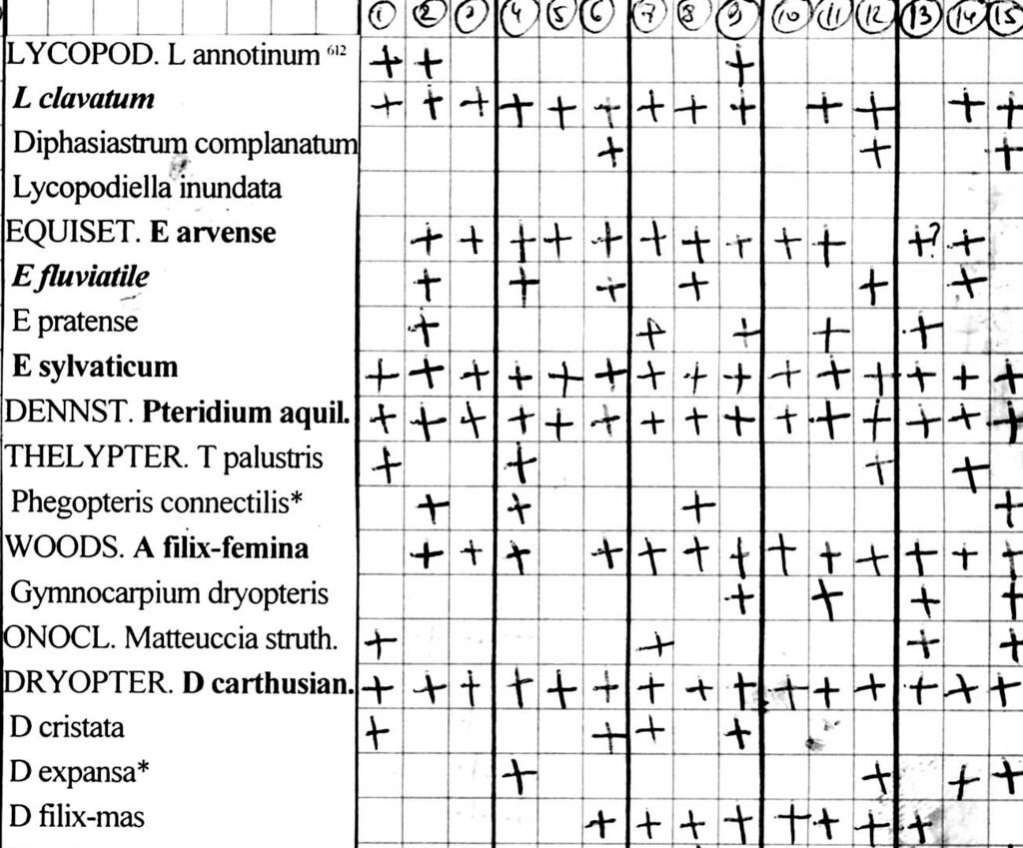
A page from a field notebook with a printed list of species used for the floristic survey of 15 grid squares in September 2012.

Usually, a floristic survey of a grid square took one day (6–9 h, sometimes up to 12 h). The track was permanently controlled using GPS in the field. Before 2018, the author used a printed spreadsheet in a field notebook with a list of the most common plants, which comprised about half of the regional flora (Fig. 4). Rarer plants were placed at the end of the list, whereas both species not identified with certainty and those of interest were collected. In 2019 and 2020, field documentation of the flora was performed using a smartphone in line with the "Flora of Russia" initiative (Seregin et al. 2020).

### Quality control

**Figure 5.**
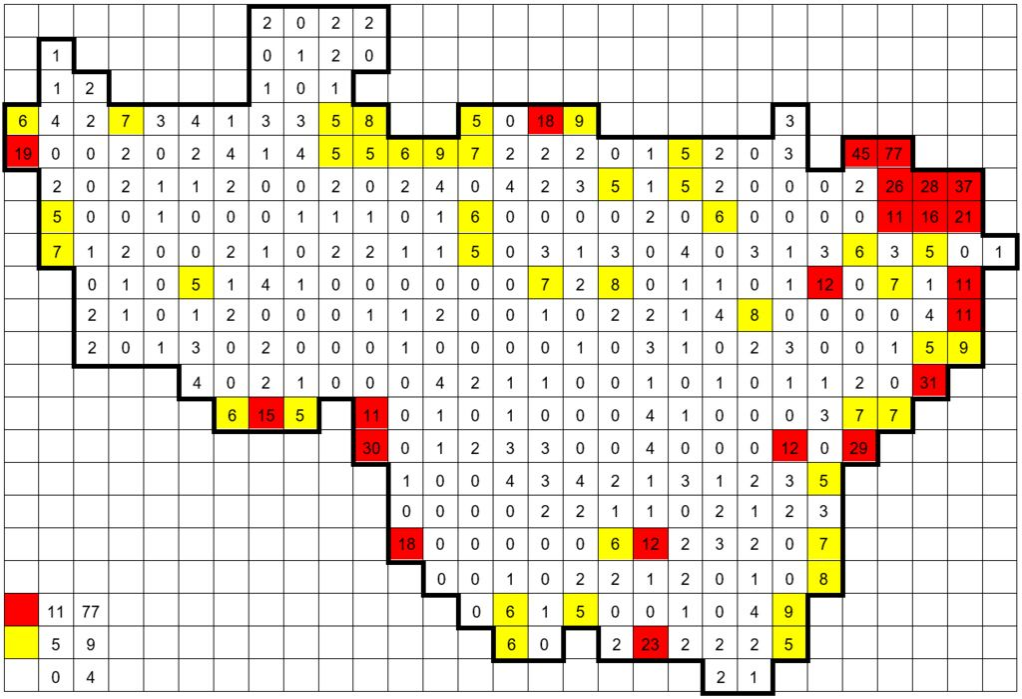
Omissions of the top-100 most recorded species.

During field surveys, we kept a record of 680 most widely distributed species on printed spreadsheets to avoid omissions of common species. Nonetheless, a map of omissions of the top-100 most recorded species (Fig. 5) suggests that some grid squares were likely under-surveyed. One can see some under-surveyed grid squares on the fringes of Vladimir Oblast (i.e. on the borders of the Region), as well as a few poorly sampled grid squares across the area. A group of red squares on the north-eastern corner shows the Balakhna Lowland (Fig. 2) with unfavourable conditions of nutrient-poor acid habitats, such as extremely dry pine forests on alluvial sands.

## Geographic coverage

### Description

**Figure 6.**
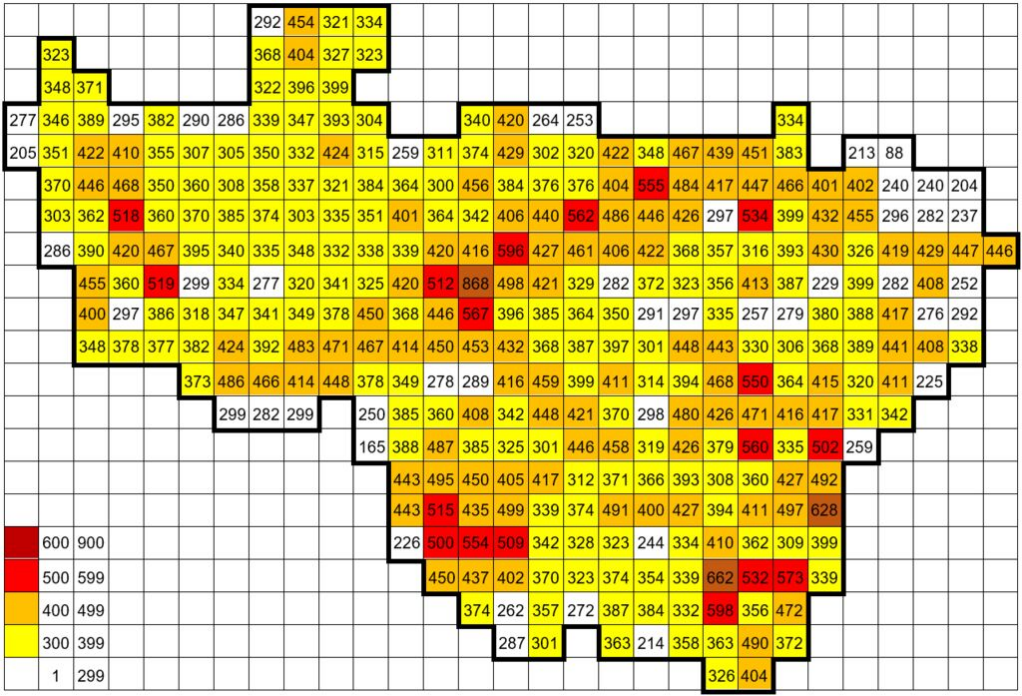
Number of records per grid (equalling the number of taxa).

Vladimir Oblast, Russia, in its administrative borders and some records from adjacent parts of the grid squares, which are only partly within the Vladimir Oblast borders. During 21 years, the area was evenly sampled, thus the number of recorded species across grid squares gives a good overview of natural patterns, rather than sampling efforts (Fig. 6). Spatial data on the vascular plant flora of Vladimir Oblast were published earlier in the form of 1,370 species distributional maps (Seregin 2012).

The second book of the series (Seregin 2014) included an analytical part of the survey. A quantitative spatial assessment at various scales, an overview of distributional patterns for common and rare species and spatial analysis of grid distributions led to recognition of the regional chorotypes (i.e. distributional species groups within the Region) and confirmed the presence of ten floristic divisions (Fig. 2).

### Coordinates

55 and 57 Latitude; 38 and 43 Longitude.

## Taxonomic coverage

### Description

A total of 1,465 vascular plant taxa–largely species, but also hybrids, microspecies, undivided genera and some uncertain species. Table 4 shows the top-100 most widely-distributed species across the grid squares, giving a general overview of the common plant species and communities. The flora of Vladimir Oblast is a typical temperate European flora dominated by some forest, meadow and ruderal species. *Erigeron canadensis* L. with 327 grid records is the most widespread alien species.

### Taxa included

**Table:** 

Rank	Scientific Name	
phylum	Tracheophyta	

## Traits coverage

### Data coverage of traits

PLEASE FILL IN TRAIT INFORMATION HERE

## Temporal coverage

### Notes

01-01-1867 through to 31-01-2021.

**Figure 7.**
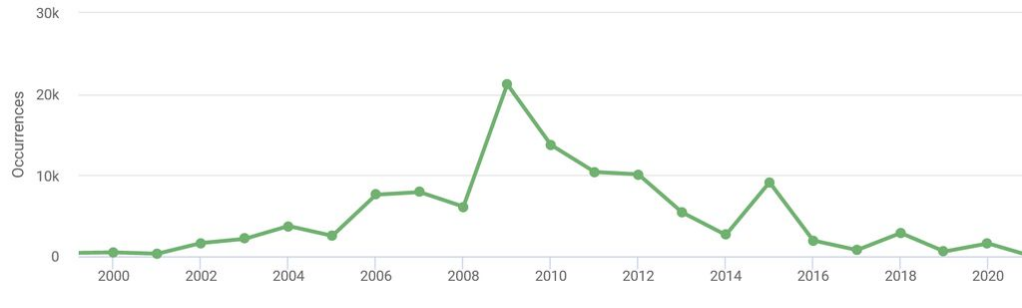
Year of the latest grid records within the dataset (Seregin 2021b).

The year of observation is clearly indicated in 113,578 grid records (87.3%). Undated records resulted from digitisation of old references and specimen records, as well as from earlier surveys during which an interval instead of a specific date was indicated. As we include only the latest grid records for each species, the number of undated records is permanently decreasing. Merely all dated records (i.e. 112,992) were made during 2000–2020. In 2009, 21,220 grid records were added into the dataset (Fig. 7).

*Carex elongata* L. (Cyperaceae) is used here as an example of a previously under-recorded species to show the recent progress in data collection (Fig. 8). This species was reported from 79 grid squares in the standard flora (Fig. 8a) (Seregin 2012). In Vladimir Oblast, *C. elongata* is a typical plant of Alnetea glutinosae communities (alder forests), which are extremely inhospitable for a researcher during the spring and summer seasons due to mosquitoes and boggy ground. Therefore, the data on this species were far from complete. Further focused surveying of this habitat during the last decade and expertise in identification of this sedge without fruits helped us to double the number of the known records published in this dataset (Fig. 8f).

By the end of 2017, many biased maps of species grid distributions were updated as a result of extensive field surveys. Thereby, the data collected during the last three years (2018 to 2020) clearly indicate further expansion of invasive or potentially invasive species (Seregin 2010, Seregin 2015). For instance, *Erigeron septentrionalis* (Fernald et Wiegand) Holub, *Epilobium tetragonum* L. agg., *Oenothera biennis* L., *Anisantha tectorum* (L.) Nevski and *Jacobaea vulgaris* Gaertn. are the most rapidly expanding aliens in the last three years (Table 5). Surprisingly, a steady growth of the grid records for common orchids like *Platanthera bifolia* (L.) Rich. and *Dactylorhiza fuchsii* (Druce) Soó is noticeable as well.

## Usage licence

### Usage licence

Other

### IP rights notes

This work is licensed under a Creative Commons Attribution (CC-BY) 4.0 License.

## Data resources

### Data package title

Flora of Vladimir Oblast, Russia: an updated grid dataset (1867-2020)

### Resource link

https://www.gbif.org/dataset/7afb26e9-aad6-47cb-a5bf-de49dc7597a4

### Alternative identifiers

7afb26e9-aad6-47cb-a5bf-de49dc7597a4, https://depo.msu.ru/ipt/resource?r=vladimir

### Number of data sets

1

### Data set 1.

#### Data set name

Flora of Vladimir Oblast, Russia: an updated grid dataset (1867-2020)

#### Data format

Darwin Core

#### Number of columns

47

#### Description

"Flora of Vladimir Oblast, Russia: an updated grid dataset (1867–2020)" contains 130,054 unique occurrences of 1,465 vascular plant taxa (species, hybrids, aggregates) from Vladimir Oblast and tiny parts of the adjacent areas. The average number of grid records increased in seven years from 363 to 380 species (Seregin 2021b).

The grid occurrences are largely based on the field studies by the author performed in 1999–2020 (121,737 records), as well as on the data extracted from relevant literature, manuscripts, herbarium collections and citizen science projects (8,317 records). An aggregation of the grid records by 342 grid squares was performed using "Event ID" field of the DarwinCore. Taxonomic backbone of the occurrence grid dataset is following Seregin (2014) which is available in GBIF as a checklist dataset (Seregin 2021c) to ensure smooth cross-linking of the names.

As of April 2021, "Flora of Vladimir Oblast, Russia: an updated grid dataset (1867–2020)" is the fourth largest dataset on vascular plants of Russia published via GBIF.

**Data set 1.** 

Column label	Column description
occurrenceID	An identifier for the occurrence. A variable constructed from a combination of two identifiers (datasetID and catalogNumber). For example, "urn:lsid:biocol.org:col:15550:02:000001".
dcterms:type	The nature or genre of the resource. A constant ("Dataset").
dcterms:modified	The most recent date-time on which the resource was changed. A constant ("2021-04-16").
dcterms:language	A language of the resource. A constant ("en" = English)
dcterms:license	A legal document giving official permission to do something with the resource. A constant ("http://creativecommons.org/licenses/by/4.0/legalcode").
dcterms:rightsHolder	A person or organisation owning or managing rights over the resource. A constant ("Moscow State University").
dcterms:accessRights	Information about who can access the resource or an indication of its security status. A constant ("Use under CC BY 4.0").
institutionID	An identifier for the institution having custody of the object(s) or information referred to in the record. A constant ("http://grbio.org/institution/moscow-state-university" for the Moscow Sate University).
collectionID	An identifier for the collection or dataset from which the record was derived. A constant ("urn:lsid:biocol.org:col:15550" for the Moscow University Herbarium).
datasetID	An identifier for the set of data. May be a global unique identifier or an identifier specific to a collection or institution. A constant ("urn:lsid:biocol.org:col:15550:02").
institutionCode	The name (or acronym) in use by the institution having custody of the object(s) or information referred to in the record. A constant ("Moscow State University").
collectionCode	The name, acronym, coden or initialism identifying the collection or dataset from which the record was derived. A constant ("MW" for the Moscow University Herbarium).
datasetName	The name identifying the dataset from which the record was derived. A constant ("Flora of Vladimir Oblast, Russia: an updated grid dataset (1867-2020)").
ownerInstitutionCode	The name (or acronym) in use by the institution having ownership of the object(s) or information referred to in the record. A constant ("Moscow State University").
basisOfRecord	The specific nature of the data record - a subtype of the dcterms:type. A variable (three terms: "Literature", "PreservedSpecimen", "HumanObservation" before translation). "Literature" was translated as "HumanObservation" following Darwin Core Type Vocabulary.
informationWithheld	Additional information that exists, but that has not been shared in the given record. A constant ("Occurrence is placed in the grid square centroid; real coordinates, habitat details and voucher information (if present) are obscured.")
dataGeneralizations	Actions taken to make the shared data less specific or complete than in its original form. A constant ("Occurrence is placed in the grid square (5.0′ lat. x 10.0′ long.) centroid. Only one record per grid per taxon is included in the dataset (normally, the latest one).")
catalogNumber	An identifier (preferably unique) for the record within the dataset or collection. A variable. For example, "000001".
recordedBy	A list (concatenated and separated) of names of people, groups or organisations responsible for recording the original occurrence. A variable.
occurrenceStatus	A statement about the presence or absence of a taxon at a location. A constant ("present").
associatedReferences	A list (concatenated and separated) of identifiers (publication, bibliographic reference, global unique identifier, URI) of literature associated with the Occurrence. A constant ("Seregin, A.P. assisted by Borovichev, E.A., Glazunova, K.P., Kokoshnikova, Y.S. and Sennikov, A.N. (2012): Flora of Vladimir Oblast, Russia: checklist and atlas. Tula. Grif i K. 620 pp. (in Russian, with English abstract). http://dx.doi.org/10.13140/RG.2.1.4544.5122/1").
eventID	An identifier for the set of information associated with an event. A variable constructed from a combination of three identifiers (grid square index from verbatimLocality, eventDate and initials of the recordedBy person). For example, "Н3/2004-10-23/APS".
year	The four-digit year in which the event occurred, according to the Common Era Calendar. A variable.
month	The ordinal month in which the event occurred. A variable.
day	The integer day of the month on which the event occurred. A variable.
eventDate	The date or interval during which an event occurred. For occurrences, this is the date when the event was recorded. A variable.
eventRemarks	Comments or notes about the event. A variable (three options: "Standard survey period 1867-1949", "Standard survey period 1950-1999", "Standard survey period 2000-2020").
higherGeography	A list (concatenated and separated) of geographic names less specific than the information captured in the locality term. A constant ("Europe | Russian Federation | Vladimir Oblast").
continent	The name of the continent in which the location occurs. A constant ("Europe").
country	The name of the country or major administrative unit in which the location occurs. A constant ("Russian Federation").
countryCode	The standard code for the country in which the location occurs. A constant ("RU").
stateProvince	The name of the next smaller administrative region than country (state, province, canton, department, region etc.) in which the location occurs. A constant ("Vladimir Oblast").
verbatimLocality	The original textual description of the place. A variable with grid square index. For example, "Grid square Е17".
locationAccordingTo	Information about the source of this location information. Could be a publication (gazetteer), institution or team of individuals. A constant ("Seregin, A.P. assisted by Borovichev, E.A., Glazunova, K.P., Kokoshnikova, Y.S. and Sennikov, A.N. (2012): Flora of Vladimir Oblast, Russia: checklist and atlas. Tula. Grif i K. 620 pp. (in Russian, with English abstract). http://dx.doi.org/10.13140/RG.2.1.4544.5122/1").
decimalLatitude	The geographic latitude (in decimal degrees, using the spatial reference system given in geodeticDatum) of the geographic centre of a location. A variable (latitude of a grid square centroid).
decimalLongitude	The geographic longitude (in decimal degrees, using the spatial reference system given in geodeticDatum) of the geographic centre of a location. A variable (longitude of a grid square centroid).
geodeticDatum	The ellipsoid, geodetic datum or spatial reference system (SRS) upon which the geographic coordinates given in decimalLatitude and decimalLongitude are based. A constant ("WGS84").
coordinateUncertaintyInMeters	The horizontal distance (in metres) from the given decimalLatitude and decimalLongitude describing the smallest circle containing the whole of the location. A constant ("7000" or an average distance between a grid square centroid and a grid square corner).
georeferencedBy	A list (concatenated and separated) of names of people, groups or organisations who determined the georeference (spatial representation) of the location. A constant ("Alexey P. Seregin").
identifiedBy	A list (concatenated and separated) of names of people, groups or organisations who assigned the Taxon to the subject. A variable (for example, "Alexey P. Seregin").
scientificName	The full scientific name, with authorship and date information, if known. A variable (for example, "*Diphasiastrum complanatum* (L.) Holub").
kingdom	The full scientific name of the kingdom in which the taxon is classified. A constant ("Plantae").
phylum	The full scientific name of the phylum or division in which the taxon is classified. A constant ("Tracheophyta").
genus	The full scientific name of the genus in which the taxon is classified. A variable (for example, "*Diphasiastrum*").
taxonRank	The taxonomic rank of the most specific name in the scientificName. A variable (three options: "Species", "Genus", "Variety").
nomenclaturalCode	The nomenclatural code (or codes in the case of an ambiregnal name) under which the scientificName is constructed. A constant ("International Code of Nomenclature for algae, fungi and plants").
taxonomicStatus	The status of the use of the scientificName as a label for a taxon. A constant ("accepted"). The taxonomy is linked to a checklist dataset (https://doi.org/10.15468/7zk2y5) that defines the concept.

## Supplementary Material

E068E13B-4F91-5DF3-99C6-568C94EDBDC8 10.3897/BDJ.9.e68046.suppl1 Supplementary material 1 A grid scheme used for "Flora of Vladimir Oblast, Russia" dataset Data type shapefile of the grid (*.kml) Brief description A grid scheme (*.kml file) used for georeferences in the "Flora of Vladimir Oblast, Russia: an updated grid dataset (1867–2020)" (https://doi.org/10.15468/hoafrr). It is based on the WGS84 datum with 342 squares with areas ranging from 94.7 km^2^ in the northernmost part to 98.2 km^2^ on the southern boundary (5′ lat. × 10′ long.). Each occurrence is linked to the corresponding grid square centroid. Cyrillic letters were used to designate 21 rows from north to south, while numbers were used to indicate the squares within the rows from west to east. The northern border of the northernmost row А follows 56°50′N, while the southern border of the southernmost row Х follows 55°05′N, the western border of the squares Г1 and Д0 follows 38°10′E, while the eastern border of the square З28 follows 43°00′E.
This file is also available on Zenodo (https://doi.org/10.5281/zenodo.4724913).
An earlier version of the grid is available on Google Maps as a kml file at https://maps.google.com/maps/ms?msid=200284766630468455543.000462414ec0fd70a9c6f&msa=0 File: oo_549170.kml https://binary.pensoft.net/file/549170 A.P. Seregin

## Figures and Tables

**Figure 8a.**
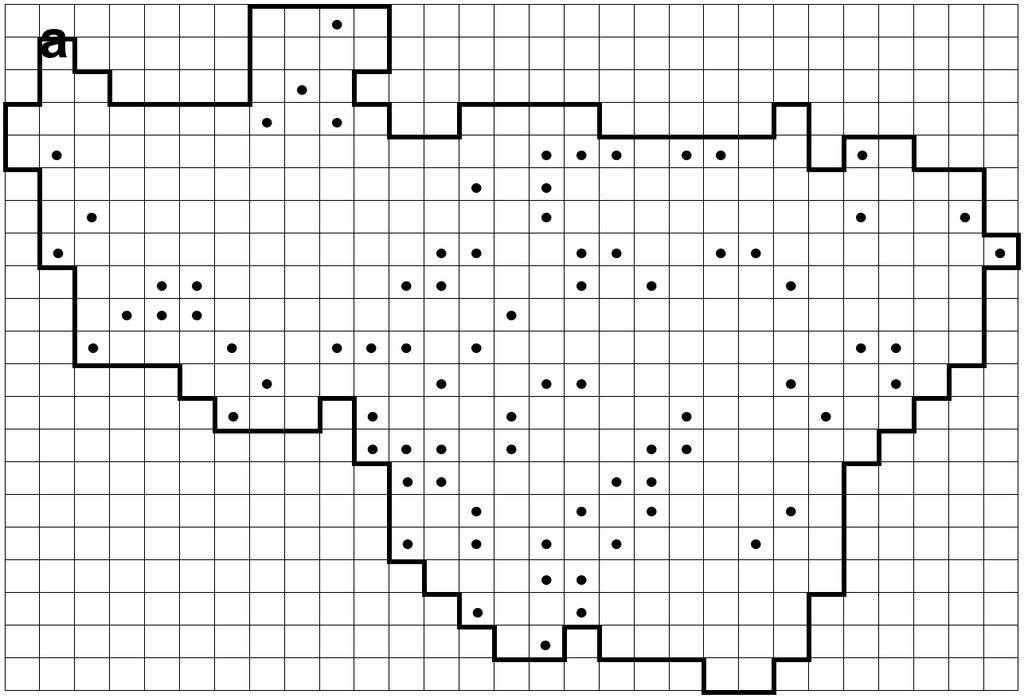
By the end of 2011 (79 grid records). Same data as published in the standard flora (Seregin 2012)

**Figure 8b.**
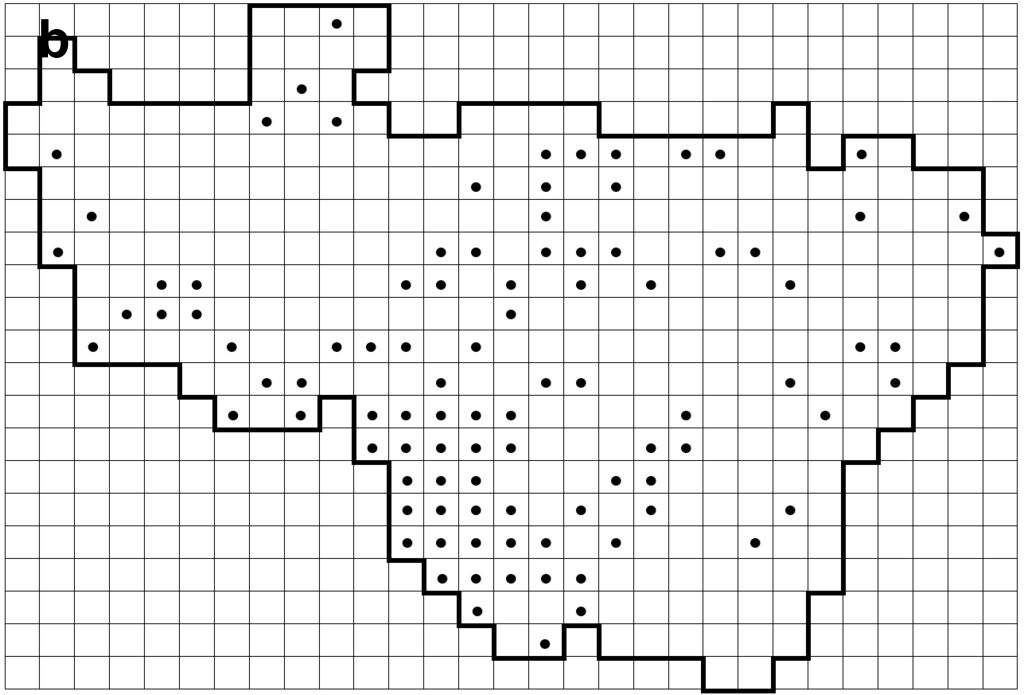
By the end of 2012 (97 grid records)

**Figure 8c.**
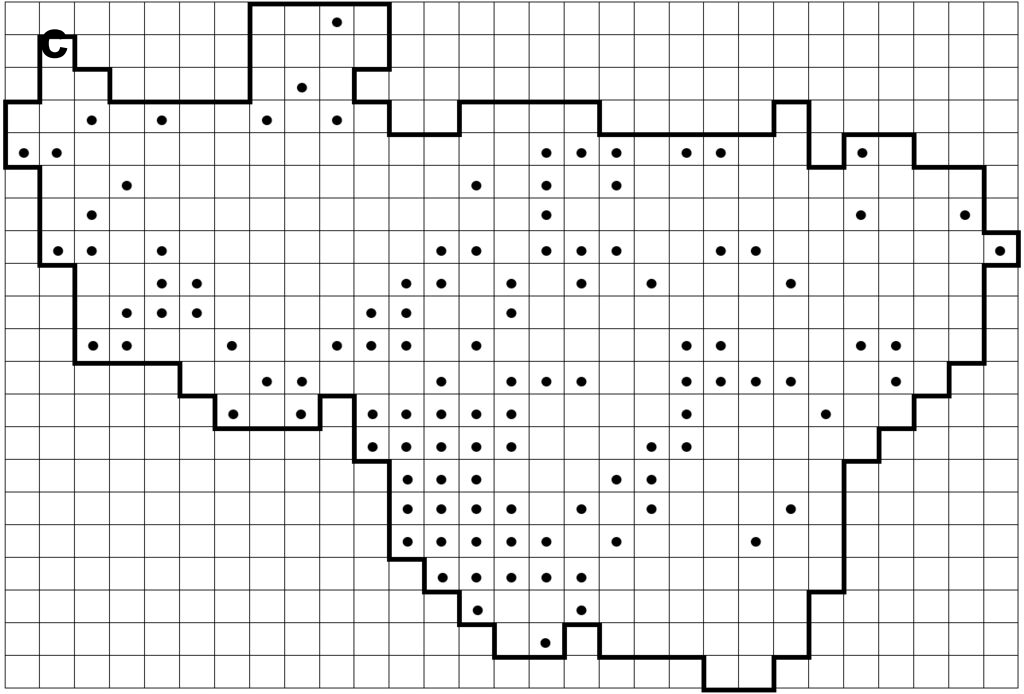
By the end of 2014 (112 grid records)

**Figure 8d.**
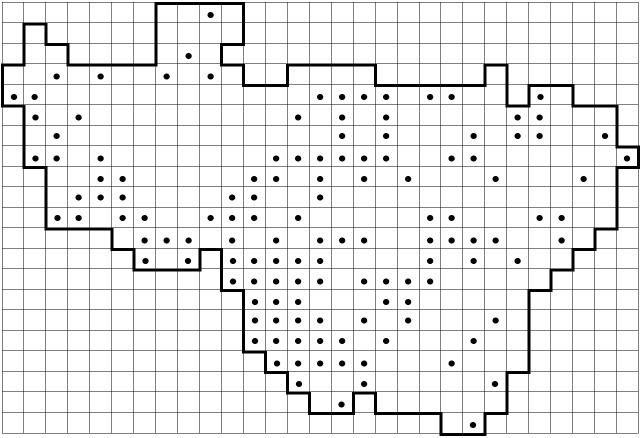
By the end of 2015 (130 grid records)

**Figure 8e.**
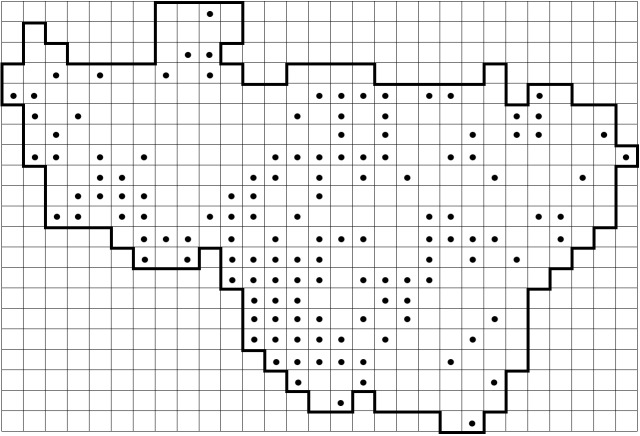
By the end of 2017 (133 grid records)

**Figure 8f.**
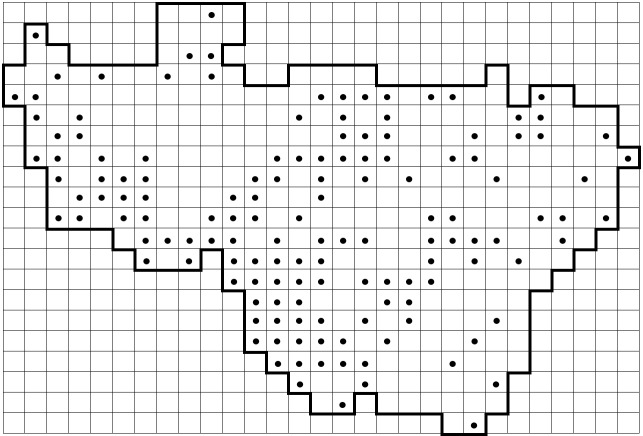
By the end of 2020 (140 grid records). Current dataset

**Table 1.** The growth of the dataset during 2011–2020. The earlier version of the dataset with 118,231 grid records (as of late 2011) was used for the map production in the standard flora (Seregin 2012).

End of the year	Number of records	Number of grid squares	Average number of records per grid square
2011	118,231	337	350.8
2012	120,854	337	358.6
2013	123,049	339	363.0
2014	124,100	339	366.1
2015	126,682	339	373.7
2016	127,245	339	375.4
2017	127,415	339	375.9
2018	128,966	342	377.1
2020	130,073	342	380.3

**Table 2.** Top-10 datasets by the number of records on the biodiversity of Russia published in GBIF (as of 19 April 2021).

Rank	Dataset	Reference	Number of records
1	iNaturalist Research-grade Observations	Ueda (2021)	1,247,040
2	Moscow University Herbarium (MW)	Seregin (2021a)	659,565
3	RU-BIRDS.RU, Birds observations database from Russia and neighbouring regions	Ukolov et al. (2019)	433,635
4	EOD - eBird Observation Dataset	Levatich and Ligocki (2020)	282,227
5	Geographically-tagged INSDC sequences	European Nucleotide Archive (EMBL-EBI) (2019)	195,451
6	Locations of plants on dot distribution maps in the Flora of Siberia (Flora Sibiraea, 1987–1997)	Artemov and Egorova (2021)	169,854
7	Flora of Vladimir Oblast, Russia: an updated grid dataset (1867-2020)	Seregin (2021b)	130,054
8	Finnish Floristic Database (Finnish Museum of Natural History Collections)	Lampinen and Laiho (2021)	106,396
9	Birds of Northern Eurasia	Karyakin et al. (2020)	86,992
10	Chronicle of Nature - Phenology of Plants of Zhiguli Nature Reserve	Kiseleva (2021)	86,524

**Table 3.** Top-10 datasets by the number of records published in GBIF by the Russian institutions (as of 19 April 2021).

Rank	Dataset	Reference	Number of records
1	Moscow University Herbarium (MW)	Seregin (2021a)	1,025,148
2	RU-BIRDS.RU, Birds observations database from Russia and neighbouring regions	Ukolov et al. (2019)	468,333
3	Locations of plants on dot distribution maps in the Flora of Siberia (Flora Sibiraea, 1987–1997)	Artemov and Egorova (2021)	169,854
4	Flora of Vladimir Oblast, Russia: an updated grid dataset (1867-2020)	Seregin (2021b)	130,054
5	Birds of Northern Eurasia	Karyakin et al. (2020)	90,996
6	Chronicle of Nature - Phenology of Plants of Zhiguli Nature Reserve	Kiseleva (2021)	86,524
7	MHA Herbarium: collections of vascular plants	Seregin and Stepanova (2021)	78,193
8	Chronicle of Nature - Phenology of Plants of FSE Zapovednoe Podlemorye	Bukharova (2021)	54,792
9	Birds and Mammals Collections of the Zoological Museum of M.V. Lomonosov Moscow State University	Lomonosov Moscow State University (2018)	54,120
10	CRIS dataset	Community of CRIS and Melechin (2019)	54,054

**Table 4.** Top-100 most recorded species of Vladimir Oblast flora (318+ grid records).

**Rank**	**Species**	**Number of grid squares**
1	*Betula pendula* Roth	342
2	*Chamaenerion angustifolium* (L.) Scop.	342
3	*Hieracium umbellatum* L.	342
4	*Lysimachia vulgaris* L.	342
5	*Plantago major* L.	342
6	*Populus tremula* L.	342
7	*Sorbus aucuparia* L.	342
8	*Achillea millefolium* L.	341
9	*Calamagrostis epigejos* (L.) Roth	341
10	*Deschampsia cespitosa* (L.) P. Beauv.	341
11	*Tanacetum vulgare* L.	341
12	*Artemisia vulgaris* L.	340
13	*Polygonum aviculare* L. agg.	340
14	*Ranunculus repens* L.	340
15	*Salix cinerea* L.	340
16	*Trifolium repens* L.	340
17	*Angelica sylvestris* L.	339
18	*Equisetum arvense* L.	339
19	*Solidago virgaurea* L.	339
20	*Urtica dioica* L.	339
21	*Galium mollugo* L.	338
22	*Phleum pratense* L.	338
23	*Pinus sylvestris* L.	338
24	*Potentilla argentea* L.	338
25	*Rubus idaeus* L.	338
26	*Dryopteris carthusiana* (Vill.) H.P. Fuchs	337
27	*Pimpinella saxifraga* L.	337
28	*Poa annua* L.	337
29	*Salix caprea* L.	337
30	*Taraxacum officinale* Wigg. agg.	337
31	*Trifolium pratense* L.	337
32	*Veronica chamaedrys* L.	337
33	*Vicia cracca* L.	337
34	*Chenopodium album* L. agg.	336
35	*Convallaria majalis* L.	336
36	*Linaria vulgaris* Mill.	336
37	*Bromopsis inermis* (Leyss.) Holub	335
38	*Festuca rubra* L.	335
39	*Fragaria vesca* L.	335
40	*Frangula alnus* Mill.	335
41	*Prunella vulgaris* L.	335
42	*Cirsium setosum* (Willd.) Besser	334
43	*Galium palustre* L.	334
44	*Potentilla anserina* L.	334
45	*Cerastium holosteoides* Fr.	333
46	*Knautia arvensis* (L.) Coult.	333
47	*Leontodon autumnalis* L.	333
48	*Quercus robur* L.	333
49	*Stellaria graminea* L.	333
50	*Alisma plantago-aquatica* L.	332
51	*Athyrium filix-femina* (L.) Roth	331
52	*Hypericum maculatum* Crantz	331
53	*Hypericum perforatum* L.	331
54	*Elytrigia repens* (L.) Desv. ex Nevski	330
55	*Equisetum sylvaticum* L.	330
56	*Picea abies* (L.) H. Karst.	330
57	*Trifolium medium* L.	330
58	*Tussilago farfara* L.	330
59	*Anthoxanthum odoratum* L.	329
60	*Dactylis glomerata* L.	329
61	*Lemna minor* L.	329
62	*Rubus saxatilis* L.	329
63	*Tripleurospermum inodorum* (L.) Sch. Bip.	329
64	*Campanula patula* L.	328
65	*Scirpus sylvaticus* L.	328
66	*Anthriscus sylvestris* (L.) Hoffm.	327
67	*Artemisia absinthium* L.	327
68	*Bidens tripartita* L.	327
69	*Erigeron canadensis* L.	327
70	*Filipendula denudata* (J. Presl et C. Presl) Fritsch	327
71	*Phalaroides arundinacea* (L.) Rausch.	327
72	*Viburnum opulus* L.	327
73	*Agrostis capillaris* L.	326
74	*Centaurea jacea* L.	325
75	*Glechoma hederacea* L.	325
76	*Heracleum sibiricum* L.	325
77	*Juncus tenuis* Willd.	325
78	*Mentha arvensis* L.	325
79	*Juncus bufonius* L.	324
80	*Arctium tomentosum* Mill.	323
81	*Leucanthemum vulgare* Lam.	323
82	*Poa angustifolia* L.	323
83	*Ranunculus acris* L.	323
84	*Aegopodium podagraria* L.	322
85	*Capsella bursa-pastoris* (L.) Medik.	322
86	*Malus domestica* Borkh.	322
87	*Prunus padus* L.	322
88	*Rorippa palustris* (L.) Besser	322
89	*Viola canina* L.	322
90	*Salix myrsinifolia* Salisb.	321
91	*Typha latifolia* L.	321
92	*Alchemilla* L. (multiple species)	319
93	*Cirsium vulgare* (Savi) Ten.	319
94	*Juncus effusus* L.	319
95	*Lycopus europaeus* L.	319
96	*Vicia sepium* L.	319
97	*Glyceria fluitans* (L.) R. Br.	318
98	*Salix triandra* L.	318
99	*Schedonorus pratensis* (Huds.) P. Beauv.	318
100	*Silene pratensis* (Rafn) Godr. et Gren.	318

**Table 5.** Growth in the number of grid records during the last three years (2017 vs. 2020) across Vladimir Oblast. Presumable causes of the data growth include true **expansion** of the alien species across the region; **earlier under-recording** of species from some habitats (such as alder forests, nutrient-poor meadows, flood plains etc.); **life cycle of some orchids** when they can be abundant or completely invisible from year to year; or **short life cycle** of spring plants.

Species	Cause of the data growth	Records by the end of 2017	Records by the end of 2020	New grid records (2017 vs. 2020)
*Erigeron septentrionalis* (Fernald et Wiegand) Holub	expansion	217	232	15
*Platanthera bifolia* (L.) Rich.	orchid's life cycle	185	197	12
*Epilobium tetragonum* L. agg.	expansion	110	122	12
*Oenothera biennis* L.	expansion	31	43	12
*Anisantha tectorum* (L.) Nevski	expansion	26	38	12
*Jacobaea vulgaris* Gaertn.	expansion	223	234	11
*Cardamine dentata* Schult.	earlier under-recording	71	82	11
*Paris quadrifolia* L.	earlier under-recording	200	210	10
*Dactylorhiza fuchsii* (Druce) Soó	orchid's life cycle	161	171	10
*Dianthus barbatus* L.	expansion	76	86	10
*Corydalis solida* (L.) Clairv.	short life cycle	70	80	10
*Impatiens glandulifera* Royle	expansion	41	51	10
*Rumex thyrsiflorus* Fingerh.	earlier under-recording	271	280	9
*Sagittaria sagittifolia* L.	earlier under-recording	172	181	9
*Lamium album* L.	expansion	32	41	9
*Myosotis sylvatica* Ehrh. ex Hoffm.	expansion	13	22	9

## Footnotes

*1 eBird dataset (Levatich and Ligocki 2020) partly attributed to Russia is exluded.
